# Online vs. Supervised Training in Relieving Urinary Incontinence and Diastasis Recti Abdominis in Early Postpartum

**DOI:** 10.3390/jcm13247730

**Published:** 2024-12-18

**Authors:** Sabina Tim, Agnieszka Mazur-Bialy

**Affiliations:** Department of Biomechanics and Kinesiology, Faculty of Health Science, Jagiellonian University Medical College, Skawińska 8, 31-066 Krakow, Poland; sabina.tim@uj.edu.pl

**Keywords:** postpartum, pelvic floor disorders, urinary incontinence, diastasis recti, pelvic floor muscle exercises, physiotherapy, telerehabilitation

## Abstract

**Background/Objectives**: The postpartum period is marked by numerous physical changes, often leading to pelvic floor disorders (PFD) such as urinary incontinence (UI) and diastasis recti abdominis (DRA). This study aimed to assess the occurrence of UI and DRA in postpartum women and evaluate the effectiveness of physiotherapy in managing UI and DRA. **Methods**: A total of 396 women, between the 3rd and 5th postpartum day, were randomized into three groups: control (GrCon), online exercise group (GrOnl), and supervised group (GrSup). GrCon received only education, whereas GrOnl and GrSup had three sessions with exercises with physiotherapist—online or supervised. Data were collected through questionnaires, ICIQ FLUTS, body posture assessments, and DRA measurements. **Results**: The results showed a significant reduction in UI and DRA symptoms across all groups, with the GrSup group showing the greatest improvement. UI symptoms decreased from 49% during pregnancy to 36.9% postpartum, with GrSup women reporting fewer urological complaints compared to the GrOnl and GrCon groups (*p* < 0.001). DRA incidence dropped from 76.2% in the early postpartum days to 23.4% at six weeks, with GrSup showing the lowest rates (9.8%). Notably, supervised physiotherapy resulted in a higher frequency (94.7%) and correct performance of PFME (72.2%) compared to the online and control groups. **Conclusions**: The study concludes that supervised physiotherapy is more effective than online sessions in managing postpartum UI and DRA, emphasizing the importance of guided exercise for better outcomes.

## 1. Introduction

The postpartum period brings significant emotional, psychological, and physical changes [1]. Pregnancy-related factors such as increased intra-abdominal pressure from the growing fetus, hormonal changes like elevated relaxin and progesterone that soften connective tissues, and genetic collagen abnormalities can all contribute to pelvic floor dysfunction (PFD) such as urinary incontinence (UI) and diastasis recti abdominis (DRA) [2]. UI, defined as involuntary urine leakage [3], is commonly triggered by activities that increase intra-abdominal pressure, such as coughing or exercise [4]. DRA, another frequent postpartum condition, results from hormonal changes and abdominal pressure during pregnancy, causing a widening of the linea alba and weakened core strength [5,6]. Although natural regeneration occurs postpartum, full recovery is not guaranteed, with persistent dysfunctions negatively impacting quality of life, limiting social and physical activities, and contributing to depression [7,8].

Exercise plays a crucial role in addressing postpartum conditions. The World Health Organization (WHO) recommends postpartum individuals engage in at least 150 min of moderate-intensity aerobic physical activity per week, along with muscle-strengthening exercises, stretching, and reducing sedentary behaviors [9]. Regular physical activity during this period has been shown to reduce depressive symptoms, improve cardiovascular health, and aid recovery from PFD [10]. Therapeutic interventions, such as pelvic floor muscle training (PFME) is the first-line treatment for UI, significantly improving muscle strength, symptoms, and quality of life when supervised [8,11,12]. Physiotherapy is the preferred conservative treatment for DRA, incorporating exercises, manual therapy, and kinesiotaping, although standardized protocols remain underdeveloped [1,11].

Despite these benefits, postpartum women face challenges such as fatigue, caregiving demands, and disrupted routines, which often limit participation in in-person programs. [13,14,15]. Digital solutions like telerehabilitation offer promising alternatives, with real-time video conferencing improving pelvic floor strength and quality of life. Studies show high satisfaction and adherence to such methods, though research on their long-term effectiveness in postpartum populations is limited [16,17,18].

Early initiation of postpartum exercise is essential, as delaying interventions until six weeks or later may miss the optimal healing period, during which tissues regenerate, muscle strength improves, and neuromuscular control is restored [19,20,21,22]. Research shows that moderate physical activity shortly after childbirth does not worsen PFD symptoms or increase associated risks but enhances early tissue healing [23]. However, most studies begin exercise protocols six weeks postpartum or later, sometimes months after delivery [19,20,21]. By this time, the tissue regeneration process is less dynamic compared to the early postpartum weeks, underscoring the importance of timely, therapeutic interventions [22].

In our study, exercises were introduced between the 2nd and 4th weeks postpartum and completed by the 6th week, targeting the critical early recovery period. To our knowledge, this is the first study to comprehensively compare supervised, online, and verbal training methods for ergonomics and pelvic floor exercises during the early postpartum phase. While previous research assessed supervised or unsupervised PFME [24] and compared online versus offline postpartum exercises using written descriptions [17], data combining early intervention with digital methods remains limited.

The aim of the study was to determine the occurrence of pelvic floor disorders among postpartum patients and to assess the effectiveness of online and supervised exercises to prevent UI and DRA.

## 2. Materials and Methods

### 2.1. Duration of the Study and Location

The study was conducted from October 2021 to April 2024 in Krakow public hospitals. Women were recruited from the maternity wards by a physiotherapist who conducted the rest of the study. The exercises took place in the Department of Biomechanics and Kinesiology, Faculty of Health Science, Jagiellonian University Medical College.

### 2.2. Approval of the Study

The Bioethics Committee of the Jagiellonian University issued a positive opinion as follows: 1072.6120.153.2020 of 25 June 2020.

### 2.3. Participants

A total of 396 women participated in the study, recruited in their 3rd–5th day postpartum. There were 213 primiparous (127 women after Vaginal Delivery (VD) and 86 after Cesarean Section (CS)) and 183 multiparous women (after VD—103; after CS—80).

### 2.4. Inclusion and Exclusion Criteria

The inclusion criteria required participants to be within the 3rd to 5th day postpartum; aged over 18 years; to have provided informed consent for participation; and without the presence of comorbidities such as unsettled hypertension, heart disease, congenital musculoskeletal diseases (e.g., arthrogryposis, clubfoot, dysplasia, Ehlers-Danlos syndrome, pectus excavatum, and pectus carinatum), or depression. Individuals were excluded if they presented with comorbidities such as uncontrolled hypertension, heart disease, congenital musculoskeletal disorders (e.g., arthrogryposis, clubfoot, dysplasia, Ehlers-Danlos syndrome, pectus excavatum, pectus carinatum), or depression; if they were aged under 18; were outside the 3rd to 5th day postpartum; or failed to provide informed consent for participation.

### 2.5. Randomization

**Figure 1:**
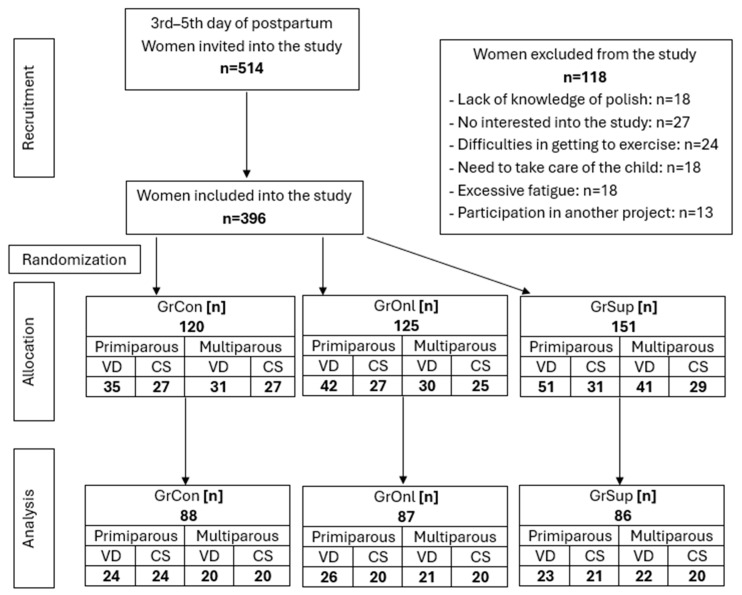
Diagram illustrating study participants by number and type of birth. VD—vaginal delivery; CS—cesarean section; GrCon—control group, women received only verbal instructions about Pelvic Floor Muscle Exercise (PFME); GrOnl—online group, women received instructions about PFME and participated in 3 online meetings during which the physiotherapist showed exercises in real time; GrSup—supervised group, women received instructions about PFME and attended 3 supervised meetings with a physiotherapist, during which the physiotherapist individually selected exercises.

Women were randomly assigned to the following groups: control group (GrCon), which received only education in proper ergonomics and instructions on how to perform pelvic floor muscle exercises (Supplementary Materials); online (GrOnl), women participated in 3 online meetings during which the physiotherapist showed exercises in real time; supervised (GrSup), where women attended 3 supervised meetings with a physiotherapist, during which the physiotherapist individually selected exercises. The exact division into groups and the number of women remaining at each stage is shown in Figure 1.

### 2.6. Questionnaires and Exams

Women who agreed to participate in the study in the hospital completed the following questionnaires: author’s and retrospective International Consultation on Incontinence Questionnaire Female Lower Urinary Tract Symptoms Long Form Module (ICIQ-FLUTS LF), which referred to the period of the last month of pregnancy. The author’s questionnaire included questions about the current delivery, pregnancy symptoms, and pelvic floor complaints. ICIQ FLUTS LF is used to assess urological symptoms in women. It contains 18 items, and each of them is assigned two questions. The first of the questions refers to the presence of a given symptom and its severity, while the second question determines the degree to which a given symptom is bothersome according to a scoring from 0 (not at all) to 10 (very much). For each question about a given symptom, women can receive from 0 (no problem) to 4 points (very big problem). The greater the sum of answers in the entire questionnaire, the greater the severity of urological problems [25]. Additionally, body posture, muscle, and DRA examinations were performed. The individual’s posture was visually assessed from the front and side while she maintained a natural stance. The patient looked straight ahead with arms relaxed and wore no clothing, shoes, or socks for clarity. The therapist observed from about 1 m away and palpated bones during the posterior assessment. Trigger Point (TrP) examination was applied to the most common muscle points. Pain was rated on a VAS scale from 0 (no pain) to 10 (unbearable pain). DRA assessment was carried out when the patient was lying supine with knees bent and lifted her head while exhaling. The therapist palpated the gap between the rectus abdominis muscles at three specific points. DRA was noted if the gap exceeded two finger widths at any point.

### 2.7. Interventions

All women after completing the examination and questionnaires in the hospital received individual information from a physiotherapist on the ergonomics of daily activities and pelvic floor exercises. Detailed instructions can be found in Supplementary Materials.

After 2 weeks (after VD) or 4 weeks (after CS) after delivery, the physiotherapist contacted the women from GrOnl and GrSup to arrange exercises. Women from GrOnl who responded to the message received an electronic link for classes. In order to be able to correct the exercises performed, a limit of 3 participants were introduced during one class. There were 3 practice meetings, at least 5 days and a maximum of 10 days apart. All meetings took place within the first 6 weeks of the postpartum period to take advantage of the pelvic floor tissue regeneration period and activate its structures [22]. A different set of exercises was performed at each meeting. For technical reasons, the exercises were previously recorded and were played back at the meeting, while all instructions regarding exercises were transmitted in real time. During the first meeting, basic instructions for everyday life were recalled. The exercises were aimed at teaching diaphragm breathing patterns, activating the gluteal muscles, correcting pelvic positioning, and progressing to pelvic floor muscle exercises. Each meeting lasted approximately 30 min. Additionally, after the exercises, women could ask questions.

In GrSup, three training sessions were planned for each woman. The break between subsequent meetings lasted a minimum of 5 days and a maximum of 10 days. Likewise, in GrOnl, all the meetings took place within the first 6 weeks of the postpartum period. The meetings were held in a group of a maximum of 3 people, which allowed proper control and supervision of the physiotherapist. Each meeting lasted approximately 60 min. A different set of exercises was conducted at each meeting. The first classes took place mainly in the supine position, where the focus was on learning diaphragm breathing and activating the transverse abdominis. Pelvic floor muscles were then activated indirectly by exercises of adductors of the thighs and buttocks. The main part of the classes ended with the direct activation of pelvic floor muscles. The second meeting focused on activating the transversus abdominis muscle and the rest of the postural muscles in higher positions. However, in the last meeting, elements of functional training were incorporated, which were intended to imitate movements used in everyday activities. The description of the online and supervised exercise protocol according to CERT guidelines can be found in Table 1. Detailed training and education protocols are provided in Supplementary Materials.

### 2.8. Follow Up

**Figure 2:**
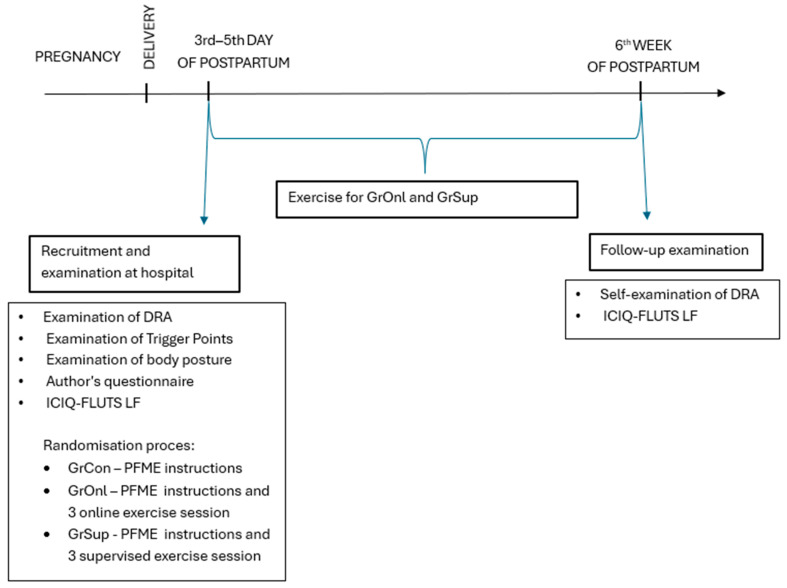
Timeline of the successive stages of the study, including the tests in each stage; DRA—diastasis recti abdominis; GrCon—control group; GrOnl—online group; GrSup—supervised group; PFME—pelvic floor muscle exercise; ICIQ FLUTS LF—International Consultation on Incontinence Questionnaire Female Lower Urinary Tract Symptoms Long Form Module.

In the 6th week of the postpartum period, all women were sent a link to a survey that included questions about pelvic floor muscle training, ICIQ FLUTS LF, and the self-examination of DRA. Each woman in the hospital received instructions on how to perform a test of the diastasis of the rectus abdominis muscles. In addition, the link that the women received with the questionnaires contained a picture and verbal instructions for performing a self-examination of DRA. The course of the different phases of the study, including the exams and questionnaires carried out in each phase, are presented in Figure 2.

### 2.9. Statistical Analysis

Statistical analysis was conducted using IBM SPSS Statistics 29. The Shapiro–Wilk test checked for normal distribution. ANOVA was applied for variables that followed a normal distribution, while the Chi-squared test was used for comparing qualitative variables. The Bonferroni method was employed for post hoc analysis, with a significance threshold set at α < 0.05.

## 3. Results

Among the patients in the different groups to which they were assigned in terms of size, age, weight, and birth weight of the child, significant differences were observed only in age. Multiparous women in each group were older than primiparous women. No significant differences were observed in the child’s weight, BMI, and child’s birth weight. Detailed characteristics of the groups are presented in Table 2.

Before pregnancy, 10.7% of women reported UI. During pregnancy, as many as 49% women reported symptoms of UI, while by the 6th week postpartum, the percentage of women with UI had dropped to 36.9%. A statistically significant relationship was found in the occurrence of leakage urine during physical activity, coughing, or sneezing, where it affected 6.7% primiparous and 42.3% multiparous (*p* = 0.001) women before pregnancy.

The severity of urological symptoms during the last month of pregnancy was greater than 6 weeks after delivery and dropped from 5.16 ± 0.26 to 2.69 ± 0.29 (*p* < 0.001). Nevertheless, women in GrSup, 6 weeks after delivery, reported less urological complaints than women in GrOnl and GrCon (GrSup: 2.49 ± 0.42; GrOnl: 2.78 ± 0.33; GrCon: 2.82 ± 0.49; *p* < 0.001).

The greatest benefits of the physiotherapy procedure were achieved in primiparous women after CS, where in GrSup, they were observed to have smaller urological symptoms than women in GrCon (*p* = 0.03). Differences in symptoms were also found among multiparous women after VD. Less severity of symptoms was observed after both online and supervised instruction compared to no intervention in GrCon (GrCon-GrOnl, *p* = 0.019; GrCon-GrSup, *p* = 0.002). However, it was noticed that in the 6th week of postpartum, the urological symptoms varied depending on the type and number of deliveries and the intervention (*p* = 0.049). In GrCon, multiparous women after VD had more symptoms than multiparous women after CS (*p* = 0.002). More details are given in Table 3.

The difference in urological points obtained in ICIQ FLUTS LF between the period of the last month of pregnancy and the 6th week of the postpartum period was also calculated, and it was noticed that a statistically greater improvement between this period was reported by women whose child’s birth weight was higher (*p* = 0.042). For women in whom the difference in urological points was the largest, the children’s birth score was 3487.33 ± 560.79, and in women with the smallest difference it was 3302.92 ± 573.69. It was also noted that 56.5% of women in whom DRA was diagnosed during pregnancy also experienced a greater increase in urological symptoms compared to women in whom DRA was not diagnosed during pregnancy (31.7%; *p* = 0.024). Women who had greater pain in the rectus femoris (2.71 ± 0.43) in the first days of the postpartum period had less improvement in urologic symptoms than women with less pain (1.37 ± 0.26; *p* = 0.009). No other dependencies were found.

Only 15.7% of women declared that they performed pelvic floor muscle exercises (PFME) before pregnancy. During pregnancy, more women started to perform these exercises, 52.1%, while the highest number of women declared that they performed the exercises after giving birth, 79.1%; however, only 45.9 per cent of them performed them correctly.

Performing PFME in the 6th week of postpartum was declared by most women from GrSup (94.7%), followed by GrOnl (79.3%), and GrCon (62.1%), (*p* = 0.001; Con-Onl *p* < 0.05; Con-Sup *p* < 0.05; Onl-Sup *p* < 0.05). In GrCon, no significant statistical differences were found in the frequency of PFME between primiparous women and the type of delivery (*p* = 0.579). However, significant differences in the frequency of PFME were found in GrCon between multiparous women after VD (70% of women exercising) and multiparous women after CS (50% of women exercising; *p* = 0.035). Between GrOnl and GrSup, no significant differences were found in the frequency of exercises depending on the type and number of deliveries, where in GrOnl, the percentage of exercising women ranged from 71.4% to 85%, and in GrSup, from 90% up to 96%.

Stationary physiotherapy treatment showed a statistically significant increase in the frequency of PFME among primiparous women after VD (*p* = 0.015) and CS (*p* = 0.04) and multiparous women after VD (*p* = 0.048) and CS (*p* = 0.006). The frequency of correctly performed PFME was lower in each of the studied groups compared to the frequency of performing the exercises. It was observed that 72.2% women who participated in GrSup performed PFME correctly, and incorrect exercise patterns most often occurred in GrCon, where only 20.4% performed PFME correctly (*p* = 0.001; (Con-Onl)-Sup *p* < 0.05). Stationary physiotherapy treatment showed a statistically significant increase in the frequency of correct performance of PFME among primiparous women after VD (*p* < 0.001) and CS (*p* = 0.005) and multiparous women after VD (*p* = 0.004) and CS (*p* < 0.001). No significant differences were found in the frequency of correct performance of PFME depending on the type and number of deliveries in GrCon (*p* = 0.358) and GrSup (*p* = 0.717). A statistically significant difference in the correctness of PFME was found in GrOnl between primiparous women after VD and CD (*p* = 0.018) and between primiparous women and multiparous women after CS (*p* = 0.034), where primiparous women after CS performed PFME correctly in most cases. More details are given in Table 4.

No dependencies were found that would determine the performance and correctness of PFME, only the group to which the women were assigned was significant.

In the first days of the postpartum period, DRA was noted among 76.2% of patients. Significant differences in the frequency of DRA in the first days of the postpartum period were observed between women after VD and CS. DRA in the first days of the postpartum period occurred in 92.2% of women after CS and 58.7% of women after VD (*p* = 0.001).

DRA in the 6th week postpartum decreased compared to the first days of the postpartum period (*p* < 0.001). It was observed that 6 weeks after delivery, DRA was present in 23.4% of women after CS compared to 10.5% of women after VD (*p* = 0.005). However, supervised exercise had the greatest impact on reducing the occurrence of DRA. In GrSup, in the 6th week of the postpartum period, the DRA was present only in 9.8% of women in GrOnl in 33.3% of patients, and in GrCon among 27.3% of women (*p* < 0.001). In GrSup, after 6 weeks after giving birth, DRA was not observed in any of the women giving birth vaginally, that is, in either among primiparous women (*p* = 0.02) and multiparous women (*p* = 0.03). Table 5 presents the results.

The DRA width in the first days of postpartum was larger in multiparous women (3.23 ± 0.14 cm) than in primiparous women (2.90 ± 0.11 cm; *p* = 0.023). In addition, the DRA width in the first days of postpartum was larger in women after CS (3.72 ± 0.12 cm) than after VD (2.44 ± 0.11 cm; *p* < 0.001). No significant differences were found between DRA width and GrCon, GrOnl, and GrSup (*p* > 0.05). However, in the 6th week of the postpartum period, it was observed that the width of the DRA was still larger in women after CS (1.62 ± 0.05 cm) than after VD (1.33 ± 0.06 cm; *p* < 0.001). In the 6th week of the postpartum period, no significant differences were found between the DRA width and the number of deliveries (*p* > 0.05). Nevertheless, women from GrSup had the smallest DRA width (1.28 ± 0.05 cm) compared to women from GrOnl (1.61 ± 0.08 cm) and GrCon (1.53 ± 0.08 cm; *p* < 0.001). From the first days of postpartum to the 6th week postpartum, on average, women in GrSup reduced their DRA by 1.66 ± 0.11 cm, those in GrOnl by 1.49 ± 0.12 cm, and those in GrCon by 1.65 ± 0.15 cm (*p* > 0.05). No significant differences were found between the difference in DRA change and the number of deliveries (*p* > 0.05), while in women after CS, the difference in DRA width between the first days after delivery and the 6th week of the postpartum period was greater (2.08 ± 0.09 cm) than women after VD (1.17 ± 0.68; *p* < 0.001).

It was found that the greatest differences in DRA width from the first days of the postpartum period to the 6th week were observed in women whose child’s birth weight was higher (3416.38 ± 541.65 g) than in women with the smallest differences in DRA width (3208.39 ± 470.42 g; *p* = 0.027). No other dependencies found.

## 4. Discussion

Pregnancy and postpartum are critical times when many women face problems such as DRA and UI. According to their prevalence, it is critical to assess the effectiveness of exercise-based interventions in preventing and treating these conditions. The aim of this study was to assess the prevalence of UI and DRA and the effectiveness of exercise in preventing these conditions in the early postpartum period.

Early postpartum therapeutic exercises, which were introduced in our study, are essential for addressing the complex physiological changes that occur after childbirth and for facilitating healing, increasing blood supply to tissues and improving PFM function, strength, and endurance [26]. During pregnancy, hormonal shifts, including increased levels of relaxin, estrogen, and progesterone, loosen ligaments and reduce pelvic stability, weakening the PFM and spinal support [22,27]. The growing uterus disturbs biomechanics by shifting the center of gravity, increasing lumbar lordosis, thoracic kyphosis, and pelvic anteversion, and stretching the abdominal wall, all of which compromise core strength [28]. These changes, combined with increased bladder compression and urinary output, can lead to pelvic floor dysfunctions, especially stress UI [29]. Vaginal delivery exacerbates these effects through nerve damage, ligament injuries, and connective tissue disruption, further weakening pelvic support structures [30,31].

UI is a common condition that often reduces the quality of life for postpartum women [32]. Its causes are multifactorial, involving urinary, hormonal, and musculoskeletal changes affecting the PFM [33]. Stress UI is most common after childbirth, due to damage to the connective tissues supporting the bladder and urethra, and the weakening of the PFM and urethral sphincter, which reduces urethral closure pressure [33]. Factors like age, vaginal delivery, and parity also contribute to postpartum UI [32]. Our findings show that women after VD are more likely to experience UI than those after CS (43.15% vs. 29.65%), likely due to pelvic floor stretching, episiotomy, or trauma during childbirth [34]. Other studies report postpartum UI prevalence to be between 26% [35] and 55% [36]. Notably, nearly seven times more multiparous (42.3%) than primiparous women (6.7%) reported UI before their last pregnancy, highlighting pregnancy and childbirth as significant risk factors for PFD [37]. In addition to UI, up to 77% of women experience DRA in early postpartum, disrupting trunk biomechanics, pelvic stability, and body alignment, increasing the risk of lumbar and pelvic injuries [38]. DRA is more frequent in multiparous women and after CS than VD [5]. Our findings revealed that 92.2% of women after CS had DRA shortly after birth, compared to 58.7% in those who had VD, persisting at 6 weeks postpartum (23.4% vs. 10.5%). Widened separation of the abdominal muscles in DRA can lead to lumbopelvic instability, back pain, and urinary incontinence. Abdominal muscle activity during VD helps intra-abdominal pressure, facilitating childbirth [5]. These findings underscore the importance of managing DRA, particularly after CS, to improve postpartum muscle restoration.

In the first six weeks postpartum, significant healing processes occur [39]. Perineal injuries begin healing, nerve regeneration takes place, and pelvic floor muscle strength starts to improve [40]. Anatomical changes include the reduction in the hiatus area, elevation of the bladder neck [41], and restoration of posterior vaginal wall support, enhancing pelvic stability [42]. Additionally, early postpartum adjustments involve the normalization of the urinary-genital function, restoration of muscle tone, hemodynamics, and metabolism [39,41,42]. Early therapeutic exercises, such as PFME and targeted strengthening routines, improve blood supply to tissues, promote healing, and restore neuromuscular control [40,43]. This facilitates faster recovery, minimizes dysfunctions like stress UI, and enhances musculoskeletal strength and endurance. These exercise interventions leverage the body’s dynamic healing processes in the early postpartum phase. Our research showed that introducing exercises in the first weeks of the postpartum period reduced urological symptoms and DRA better than education alone. However, other researchers started their exercise protocols at a later time point, after the 6-week postpartum period, which makes it difficult to compare our results with other studies [11]. On the other hand, our intervention included three meetings, while in other studies, protocols lasted from 6 [44] to even 12 weeks [45].

PFME are the gold standard for treating and preventing PFD [8]; however, there is a lack of studies evaluating early PFME in the postpartum period [46]. In this study, women received PFME instruction from an experienced physiotherapist. Research emphasizes the importance of providing such guidance during early postpartum, as supervised PFME leads to better outcomes, improved quality of life, and reduced UI symptoms compared to unsupervised exercises [24]. Sigurdardottir et al. [47] found that PFME under the supervision of a physiotherapist reduced the frequency of UI in the 6th month postpartum, and PFM strength and endurance also improved. In our study, it was noted that women who exercised under physiotherapist supervision at 6 weeks postpartum had fewer urological symptoms than women who exercised online or women who received only verbal instructions without supervision. For PFM to bring the best results, improving PFM functions must be performed correctly and regularly [24]. Postpartum women often face challenges with PFME, such as incorrect contraction or relaxation, highlighting the need for tailored guidance and feedback using palpation or biofeedback [48]. In this study, at 6 weeks postpartum, 78.7% of women performed PFME, with better accuracy among those supervised by physiotherapists compared to those receiving verbal-only instructions. Neels et al. [49] found that over half of postpartum women practice PFME incorrectly, often engaging other muscles or holding their breath. Our study found that approximately 60% of women made similar mistakes, including failure to isolate PFM contractions and using synergistic muscles [50]. Women supervised by physiotherapists made fewer mistakes than those with online or verbal guidance, which could have resulted in less urological symptoms postpartum.

Women in both the supervised and online exercise groups reported lower urologic symptom burdens at 6 weeks postpartum compared to the control group. However, supervised exercise resulted in the greatest reduction in DRA width. Other studies also show that online training is effective in reducing DRA compared to no intervention [17,45], which aligns with our findings. Gluppe et al. [19] found no difference in DRA reduction between women who attended specialist-led exercises and those who did not attend exercises. However, some studies showed that even a minimum of two supervised exercise sessions reduced DRA [51,52]. Notably, these interventions often started after 6 weeks postpartum. Strengthening abdominal and postural muscles is recommended to address DRA and enhance lumbopelvic stability [53]. While there is no consensus on the best protocol for DRA therapy [54], supervised training did not worsen DRA [54]. Researchers agree that improving linea alba tension is key to abdominal wall functionality, prioritizing functionality, neuromuscular control, and muscle strength over complete DRA closure [11]. These findings highlight the importance of early, targeted interventions to improve postpartum outcomes.

The postpartum period is critical for addressing conditions such as DRA and UI, with early exercise interventions playing a key role in tissue healing. Our study demonstrated that supervised physical therapy and early strengthening exercises reduced urological symptoms and DRA more effectively than education alone, highlighting the benefits of individualized guidance during this phase.

### Limitation

In this study, the sample size for each group, categorized by delivery type, number of deliveries, and intervention (supervised and online), was small. Additionally, the study did not assess PFM strength, endurance, relaxation, lift, or response to increased intra-abdominal pressure. Only urological symptoms were evaluated through a questionnaire, which does not provide a functional assessment of the PFM. Furthermore, UI was assessed at just one time point, at 6 weeks postpartum, and future studies should extend the assessment to at least one year postpartum, with multiple measurement points. Our study also did not classify the types of UI, focusing only on the presence of urine leakage. Future research should address this gap to determine the most appropriate exercises more precisely. The DRA assessment at 6 weeks postpartum was conducted by the patients themselves, which could have affected the results due to potential inaccuracies in performing the test. However, each participant received instructions on how to perform the DRA test during the initial postpartum examination and again at 6 weeks. Future studies should consider using ultrasound for a more accurate assessment. Additionally, it would be valuable to expand the evaluation of postural and PFM strength and endurance. Future research should include more objective measurements, a longer exercise duration and observation period, and additional measurement points.

## 5. Conclusions

UI and DRA are common disorders in the postpartum period. Women’s awareness of PFME is low. Education about PFME is not sufficient to encourage women to exercise and learn the correct pattern of PFME. Even though women exercise their PFM in the postpartum period, most of them exercise incorrectly. Physiotherapy is an effective method for alleviating symptoms in the area of urological disorders and DRA. Supervised physiotherapy treatment is more effective in relieving UI symptoms and DRA than the online form conducted by a qualified physiotherapist.

Based on our results, we can suggest preliminary recommendations for a standardized postpartum rehabilitation protocol. Postpartum exercises should be started as soon as possible, when the patient feels ready and there are no serious injuries. There are two possible work models, one that involves stationary meetings and one that involves online meetings. We recommend stationary work as the primary option, but when there is no possibility for face-to-face meetings with a physiotherapist, it is possible to conduct online exercises with supervision as well. PFME, especially in a supervised form, should be introduced as soon as possible to prevent PFD. Additionally, lumbo-pelvic stability strengthening training and breathing exercises engaging the diaphragm should be introduced. Exercises should be adapted to the period after childbirth, the woman’s physical fitness, and the type of delivery. We recommend starting exercises from lying down and then gradually moving to higher positions (kneeling, standing). However, new studies are needed to determine the intensity, type of exercise, and training load to determine the most effective exercise protocols for women after childbirth.

## Figures and Tables

**Table 1:** Description of the exercise session protocol to online and supervised groups according to CERT guidelines.

Item Category	Item Description	
	Online Group	Supervised Group
What: materials	Equipment: exercise mat	Equipment: exercise mat, balls, resistance bands, sensory disk
Who: provider	Master of physiotherapy	
How: delivery	Group meetings, pre-recorded exercises, real-time exercise descriptions and tips, verbal motivation, increasing exercise intensity with each session.	Supervised group, verbal instructions and show exercise, verbal motivation, progress of exercise with each session.
Where: location	Online, home	Department of Biomechanics and Kinesiology, Faculty of Health Science, Jagiellonian University Medical College
When; how much: dosage	Detailed descriptions of the exercises are in Supplementary Materials.	
Tailoring: what, how	Generic exercises, level of assessment exercises based on the available literature on postpartum exercises	
How well: planned, actual	Exercise interventions were delivered and performed as planned	

**Table 2:** Characteristics of the study group, divided into control, online, and supervised groups, as well as type and number of deliveries.

Feature	Group: Control [*n*]						Group: Online [*n*]						Group: Supervised [*n*]					
		Primiparous		Multiparous				Primiparous		Multiparous				Primiparous		Multiparous		
	Overall	VD [24]	CS [24]	VD [20]	CS [20]	*p*	Overall	VD [26]	CS [20]	VD [21]	CS [20]	*p*	Overall	VD [23]	CS [21]	VD [22]	CS [20]	*p*
Age (mean ± SD)	31.43 ± 4.59	29.83 ± 4.95 ^b^	30.58 ± 4.48 ^a^	32.25 ± 4.41 ^a^	32.58 ± 3.8 ^a^	0.02	30.91 ± 4.53	29.79 ± 4.21 ^a^	30.47 ± 3.59 ^b^	32.1 ± 4.15 ^c^	33.95 ± 3.59 ^c,d^	0.002	31.25 ± 4.22	28.44± 4.24 ^b^	31.67 ± 3.87 ^a^	31.36 ± 1.86 ^a^	33.8 ± 5.09 ^c^	0.001
Weight before pregnancy [kg] (mean ± SD)	61.99 ± 11.59	61.58 ± 11.02	63.75 ± 15.08	63.25 ± 10.05	61.74 ± 7.39	0.69	62.16 ± 10.32	62.34 ± 11.42	65.37 ± 11.27	62.8 ± 10.91	61.26 ± 10.53	0.574	62.64 ± 12.28	62.16 ± 11.74	60.05 ± 10.95	62.5 ± 7.78	62.05 ± 14.8	0.684
BMI before pregnancy (mean ± SD)	18.71 ± 3.39	18.7 ± 3.06	19.13 ± 4.29	18.91 ± 2.8	18.92 ± 2.9	0.575	18.94 ± 3.47	18.67 ± 3.27	19.87 ± 3.35	18.91 ± 2.85	18.48 ± 2.69	0.580	18.89 ± 3.55	18.77 ± 3.23	18.11 ± 3.13	18.63 ± 2.09	18.79 ± 4.19	0.726
Weight at the end of pregnancy [kg] (mean ± SD)	74.13 ± 10.81	74.33 ± 11.27	76.04 ± 13.88	74.35 ± 9.71	73.15 ± 6.96	0.973	75.18 ± 11.49	74.79 ± 11.04	77.84 ± 12.01	77.0 ± 14.77	74.73 ± 12.62	0.786	74.89 ± 11.84	73.76 ± 12.58	72.9 ± 10.83	75.31 ± 8.59	75.67 ± 13.51	0.802
BMI at the end of pregnancy (mean ± SD)	33.86 ± 10.18	34.24 ± 10.09	35.74 ± 13.05	33.56 ± 8.76	33.07 ± 5.96	0.969	35.13 ± 11.07	34.27 ± 10.56	37.64 ± 11.28	36.95 ± 14.1	34.43 ± 11.15	0.787	34.62 ± 11.46	33.66 ± 10.97	32.71 ± 9.89	34.23 ± 7.65	35.65 ± 13.38	0.802
Child’s birth weight [g] (mean ± SD)	3357.56 ± 493.66	3535.42 ± 439.16	3302.10 ± 536.14	3384.5 ± 516.62	3190.79 ± 491.56	0.248	3400.85 ± 518.89	3273.33 ± 398.79	3514.74 ± 632.87	3517 ± 595.2	3391.62 ± 522.28	0.497	3312.68 ± 500.01	3382.4 ± 423.44	3039.78 ± 536.28	3410 ± 465.48	3245.3 ± 565.47	0.127

VD—vaginal delivery; CS—cesarean section; *p*—*t* student; ^a,b,c^—Bonferroni correction. Different letters indicate statistically significant differences between primiparous women after vaginal delivery (VD) and cesarean section (CS), as well as multiparous women after VD and CS, within a single group (online, control, or supervised); *p* < 0.05.

**Table 3:** Characteristics of the total values of answers provided in the International Consultation on Incontinence Questionnaire Female Lower Urinary Tract Symptoms Long Form Module (ICIQ FLUTS LF) by group, type, and number of deliveries.

Urological Dysfunctions	Group: Control [*n*]						Group: Online [*n*]						Group: Supervised [*n*]					
	Overall	Primiparous		Multiparous		*p*	Overall	Primiparous		Multiparous		*p*	Overall	Primiparous		Multiparous		*p*
		VD [24]	CS [24]	VD [20]	CS [20]			VD [26]	CS [20]	VD [21]	CS [20]			VD [23]	CS [21]	VD [22]	CS [20]	
Occurrence of UI before pregnancy [%]	14.6	5 ^a^	10 ^a^	27.8 ^b^	5.3 ^a^	0.031	9.2	15.4 ^b^	5 ^a^	4.8 ^a^	5 ^a^	0.044	13.7	4 ^a^	4.8 ^a^	22.7 ^b^	20 ^b^	0.036
Occurrence of UI during pregnancy [%]	47.1	45.8 ^a^	41.7 ^a^	65 ^b^	35 ^a^	0.022	53.5	61.5 ^b^	40 ^a^	47.6 ^a^	45 ^a^	0.032	47.3	64 ^b^	42.9 ^a^	45.5 ^a^	50 ^a^	0.047
Occurrence of UI in 6th week postpartum [%]	34.1	33.3 ^a^	29.2 ^a^	44.5 ^b^	30 ^a^	0.048	42.5	57.7 ^b^	25 ^a^	38.1 ^a^	45 ^b^	0.043	34.1	40 ^b^	23.8 ^a^	45.5 ^b^	25 ^a^	0.037
ICIQ FLUTS LF																		
Last month of pregnancy	5.27 ± 0.48	5.16 ± 0.91 ^a^	5.92 ± 0.75 ^a^	7.1 ± 1.3 ^b^	4.4 ± 0.57 ^a^	0.017	5.13 ± 0.49	6.07 ± 1.3 ^b^	4.89 ± 0.88 ^a^	4.85 ± 0.72 ^a^	4.25 ± 0.67 ^a^	0.049	4.7 ± 0.35	4.76 ± 0.8 ^a^	3.52 ± 0.54 ^a^	4.63 ± 0.61 ^a^	5.95 ± 0.72 ^b^	0.048
6th week postpartum	2.82 ± 0.49	1.9 ± 0.45 ^a^	4.05 ± 1.2 ^b^	3.11 ± 1.07 ^a^	2.33 ± 0.67 ^a^	0.048	2.78 ± 0.33	3.16 ± 0.59	3.09 ± 0.87	2.41 ± 0.61	2.4 ± 0.63	0.136	2.49 ± 0.42	2.76 ± 0.83	2.4 ± 0.56	1.76 ± 0.58	3.0 ± 1.2	0.092
Difference	2.45 ± 0.48	4.33 ± 0.84 ^b^	2.13 ± 1.4 ^a^	4.3 ± 1.31 ^b^	2.3 ± 0.73 ^a^	0.02	2.35 ± 0.53	2.91 ± 1.08 ^a^	1.8 ± 0.95 ^b^	2.44 ± 0.82 ^a^	1.85 ± 0.32 ^b^	0.034	2.21 ± 0.46	2.0 ± 0.96 ^a^	1.12 ± 0.34 ^b^	2.87 ± 0.74 ^a^	2.96 ± 1.09 ^c^	0.028

VD—vaginal delivery; CS—cesarean section; UI—urinary incontinence; *p*—ANOVA, ^a,b,c^—Bonferroni correction. Different letters indicate statistically significant differences between primiparous women after vaginal delivery (VD) and cesarean section (CS), as well as multiparous women after VD and CS, within a single group (online, control, or supervised); *p* < 0.05.

**Table 4:** Characteristics of pelvic floor muscle exercise (PFME) by group, type, and number of deliveries.

PFME [%]	Group: Control [*n*]						Group: Online [*n*]						Group: Supervised [*n*]					
	Overall	Primiparous		Multiparous		*p*	Overall	Primiparous		Multiparous		*p*	Overall	Primiparous		Multiparous		*p*
		VD [24]	CS [24]	VD [20]	CS [20]			VD [26]	CS [20]	VD [21]	CS [20]			VD [23]	CS [21]	VD [22]	CS [20]	
Performance before pregnancy	16.7	17.4 ^a^	4.2 ^b^	20 ^a^	25 ^a^	0.026	14.7	19.2	25	14.3	15	0.346	15.9	8	14.3	27.3	25	0.076
Performance during pregnancy	45.5	58.3 ^a^	41.7 ^b^	60 ^a^	30 ^b^	0.017	45.7	65.4 ^a^	60 ^a^	38.1 ^b^	25 ^b^	0.025	51.4	68 ^a^	61.9 ^a^	54.5 ^b^	55 ^b^	0.039
Performance in 6th week postpartum	62.1	62.5 ^b^	66.7 ^a^	70 ^a^	50 ^b^	0.057	79.3	84.6	80	71.4	85	0.075	94.7	96 ^a^	95.2 ^a^	95.5 ^a^	90 ^a^	0.822
Correctness of performing exercises in 6th week postpartum	20.4	20 ^b^	12.5 ^b^	35.7 ^a^	10 ^b^	0.04	30.8	18.2 ^a^	56.3 ^b^	26.7 ^a^	29.4 ^a^	0.039	72.2	79.2	65	77.3	77.8	0.715

VD—vaginal delivery; CS—cesarean section; *p*-Chi2; ^a,b^—Bonferroni correction. Different letters indicate statistically significant differences between primiparous women after vaginal delivery (VD) and cesarean section (CS), as well as multiparous women after VD and CS, within a single group (online, control, or supervised); *p* < 0.05.

**Table 5:** Occurrence of diastasis recti abdominis (DRA) in the early postpartum days and the 6th week after delivery in relation to the group, type, and number of deliveries.

DRA	Group: Control [*n*]					*p*	Group: Online [*n*]					*p*	Group: Supervised [*n*]					*p*
	Overall	Primiparous		Multiparous			Overall	Primiparous		Multiparous			Overall	Primiparous		Multiparous		
		VD [24]	CS [24]	VD [20]	CS [20]			VD [26]	CS [20]	VD [21]	CS [20]			VD [23]	CS [21]	VD [22]	CS [20]	
Occurrence of presence in 3rd–5th day postpartum [%]	70.2	50 ^a^	87.5 ^b,c^	60 ^a,c^	95 ^b^	0.001	74.4	69.2 ^a^	90 ^a,b^	66.7 ^a^	100 ^b^	0.014	74.7	60 ^b^	100 ^a^	72.7 ^a,b^	85 ^a.b^	0.008
Occurrence of presence in 6th week postpartum [%]	21.8	12.5 ^a^	20.8 ^b^	21.1 ^b^	35 ^c^	0.02	23.5	23.1 ^a,b^	25 ^a.b^	5 ^b^	42.1 ^a^	0.039	4.7	0 ^a^	5.3 ^a^	0 ^b^	10 ^b^	0.022
Average distance between RA presence in 3rd–5th day postpartum [cm]	3.18 ± 1.63	2.13 ± 1.02 ^a^	3.71 ± 1.47 ^c^	2.78 ± 1.85 ^b^	4.23 ± 1.36 ^c^	0.026	3.09 ± 1.45	2.74 ± 1.48 ^a^	3.42 ± 1.15 ^b^	2.32 ± 1.24 ^a^	4.02 ± 1.39 ^c^	0.002	2.97 ± 1.23	2.35 ± 1.18 ^a^	3.42 ± 1.21 ^b^	2.59 ± 0.88 ^a^	3.45 ± 1.23 ^b^	0.04
Average distance between RA in 6th week postpartum [cm]	1.52 ± 0.72	1.31 ± 0.84	1.53 ± 0.71	1.61 ± 0.62	1.71 ± 0.65	0.32	1.58 ± 0.77	1.57 ± 0.86 ^a^	1.76 ± 0.57 ^a^	1.16 ± 0.69 ^b^	1.96 ± 0.68 ^c^	0.006	1.3 ± 0.53	1.03 ± 0.51 ^b^	1.36 ± 0.41 ^a^	1.27 ± 0.57 ^a^	1.47 ± 0.49 ^a^	0.012
Average difference in distance between RA from 3rd to 5th day to 6th week postpartum [cm]	1.66 ± 0.15	0.82 ± 0.13 ^a^	2.17 ± 0.23 ^b^	1.18 ± 0.32 ^a^	2.52 ± 0.31 ^b^	0.025	1.51 ± 0.12	1.18 ± 0.19 ^a^	1.66 ± 0.20 ^b^	1.16 ± 0.23 ^a^	2.06 ± 0.24 ^b^	0.014	1.67 ± 0.11	1.31 ± 0.21 ^a^	2.01 ± 0.23 ^b^	1.32 ± 0.19 ^a^	1.97 ± 0.21 ^b^	0.024

VD—vaginal delivery; CS—cesarean section; RA—rectus abdominis; *p*—Chi2; ^a,b,c^—Bonferroni correction. Different letters indicate statistically significant differences between primiparous women after vaginal delivery (VD) and cesarean section (CS), as well as multiparous women after VD and CS, within a single group (online, control, or supervised); *p* < 0.05.

## Data Availability

The raw data supporting the conclusions of this article will be made available by the authors on request.

## Supplementary Materials

The following supporting information can be downloaded at: https://www.mdpi.com/article/10.3390/jcm13247730/s1, Supplementary file: Description of exercise and daily living ergonomics.
