# Supplementary material for: Online vs. Supervised Training in Relieving Urinary Incontinence and Diastasis Recti Abdominis in Early Postpartum
Source: J Clin Med. 2024 Dec 18;13(24):7730. doi: 10.3390/jcm13247730 (PMC11677346; doi:10.3390/jcm13247730)
Supplement: Supplementary file 1 [file jcm-13-07730-s001.zip › jcm-3310513-supplementary.pdf]

Description of daily ergonomics

Action	Description
Getting out of bed	With an exhale, we bend the lower limbs one by one at the hip and knee joints. Then with an exhale, we move to lying on our side. The upper limb located at the top stabilizes the body and is located at the front at the height of the chest. On the next exhale, we move to a sitting position, simultaneously moving the torso and legs.<br>After vaginal delivery: We sit on one buttock to relieve the perineum<br>After childbirth by caesarean section: we place our hands behind us, resting the body weight on them to relieve the abdominal wall<br>On the next exhale, we move to a standing position
Micturion	We sit on the toilet with our feet hip-width apart. We do not pee in a half-squat position. Underwear, pants down to the ankles, so as not to additionally strain the muscles of the lower limbs. We maintain an upright body posture. We do not push urine, we do not accelerate its stream. We do not go to the toilet "just in case". We do not exercise the pelvic floor muscles to stop the stream of urine.
Defecation	We sit on the toilet with our feet hip-width apart, with a footrest under our feet. Underwear, trousers pulled down to the ankles, so as not to further strain the muscles of the lower limbs. We slightly round our backs. During defecation, we avoid pushing and sitting on the toilet for a long time. After defecation, we tense the muscles of the pelvic floor to restore the basic tension.
Lifting things	During the postpartum period, we avoid lifting things heavier than the current weight of the child. We position ourselves with our front on the given thing. We keep a straight spine. With an exhale, we bend our lower limbs. From a squat, we lift the given thing, holding it close to the body. We do not make twisting movements with the thing we are holding
Cough, sneezing	We maintain a straight posture. We avoid hunching or bending forward. We turn our heads to the right or left. We can cross our legs at the ankles, but we do not valgus our knees. After a cesarean section, in order to stabilize the scar, we can place our hands on the scar and gently bring its ends closer together.
Ergonomics of taking care of a child	When lifting a child, the spine should be straight and the feet should be evenly loaded. On the exhale, lift the child to your chest. When lifting, avoid twisting the torso, approach the child with your front.<br>The height of the changing table and bathtub should be adjusted to the height of the caregivers. So as not to round the spine unnecessarily.<br>When feeding, you should adopt a comfortable sitting position with a backrest, or lie on your side.

Description of exercises conducted in the online group

Outline of the first meeting			
**No.**	**Position and move**	**Number of repetitions**	**Aim**
1.	Starting position: sitting on heels, hands placed on the lower, side ribs<br>With the inhale we direct the air to the hands, feeling their expansion, with the exhale we feel the return movement under the hands.	8	Diaphragm activation
2.	Starting position: sitting on heels, the right hand from the front embraces the lower ribs, the left hand is placed on the right shoulder<br>We direct the inhalation to the right hand to feel the expansion of the lower ribs, the hand placed on the shoulder is motionless.	5 by side	Diaphragm activation
3.	Starting position: kneeling<br>Arm circles backwards.	12	Mobilization and activation of the shoulder
4.	Starting position: kneeling<br>The upper limbs are bent at 90 degrees at the elbows, with inhalation the movement of abduction of the forearms, with exhalation the return of the forearms.	12	Strengthening the back muscles
5.	Starting position: kneeling<br>Upper limbs bent at 90 degrees in elbows, elbows raised to the side to shoulder height, with inhalation raise arms upwards, with exhalation return.	12	Strengthening the shoulder muscles
6.	Starting position: lying on back, lower limbs bent<br>With an exhale, perform the movement of bringing the pubic symphysis closer to the sternum (posterior tilt of the pelvis), with an inhale, return.	20	Activation of abdominal muscles
7.	Starting position: lying on back, lower limbs bent<br>A pillow is placed between the knees, on the exhale the knees are squeezed, on the inhale relax.	12/ 2 series	Strengthening of the lower limbs, indirect activation of the pelvic floor muscles
8.	Starting position: lying on back, lower limbs bent<br>With the exhale, raise the buttocks, with the inhale, lower them.	12/ 2 series	Strengthening of the lower limbs, indirect activation of the pelvic floor muscles

9.	Starting position: lying on your back, lower limbs bent<br>With the exhale, tension, elevation of the pelvic floor muscles, with the inhale, relaxation (with the visualization of the lift; force about 20%).	12/ 2 series	Direct activation of the pelvic floor muscles
10.	Starting position: lying on the right side, lower limbs bent, support on the right forearm<br>With an exhale, raise the left knee, so as not to lift the foot, the torso is stabilized, with an inhale, lower the knee, after the series is completed, change sides.	12/ 2 series	Strengthening of the lower limbs, indirect activation of the pelvic floor muscles
11.	Starting position: lying on the right side, lower limbs bent<br>With an inhale, turn "behind the back", try to put the straight left arm behind you as far as possible, with an exhale, return, after the series is finished, change sides.	8	Stretching of the chest
12.	Starting position: lying on the right side, lower limbs extend<br>With an exhale, raise the left leg, with an inhale, lower it, after completing the series, change sides.	12/ 2 series	Strengthening of the lower limbs
13.	Starting position: cross-legged position<br>Head tilt to the right, left, back and forth.	Hold for 30 seconds	Neck relaxation
14.	Starting position: cross-legged position<br>Bend the torso to the right, then to the left.	Hold for 30 seconds	Torso relaxation
15.	Starting position: cross-legged position<br>Turn the torso to the right, then to the left.	Hold for 30 seconds	Torso relaxation
**Outline of the second meeting**			
1.	Starting position: sitting on heels, hands placed on the lower, side ribs<br>With the inhale we direct the air to the hands, feeling their expansion, with the exhale we feel the return movement under the hands.	8	Diaphragm activation
2.	Starting position: kneeling supported on hands<br>With exhalation, the movement of approaching the pubic symphysis to the sternum, with inhalation, return.	12	Activation of the pelvic floor muscles and mobilization of the pelvis and spine
3.	Starting position: kneeling supported on hands<br>Pelvic rotation to the right, then change direction. When the pubic symphysis approaches the sternum, exhale, when it recedes, inhale.	12	Activation of the pelvic floor muscles and mobilization of the pelvis and spine

4.	Starting position: kneeling supported on hands<br>With exhalation, tighten and elevate the pelvic floor muscles, with inhalation, relax (strength approx. 30%).	12/ 2 series	Direct activation of the pelvic floor muscles
5.	Starting position: lying on back, lower limbs bent, upper limbs raised up<br>With an exhale, bring the right knee to the chest and the left hand to the navel, with an inhale, return to the starting position. After the series is complete, change sides.	12/ 2 series	Strengthening of abdominal muscles
6.	Starting position: lying on back, lower limbs bent, right lower limb raised to 90 degrees and bent in the knee and hip joints.<br>With an exhale, abduct the right lower leg, with an inhale, return to the starting position, after the completed series, change sides.	12/ 2 series	Strengthening of abdominal muscles
7.	Starting position: kneeling supported on hands, right upper limb raised to the side<br>With exhalation, raise the straightened left lower limb, with inhalation, return. After finishing the series, change sides.	12/ 2 series	Strengthening the abdomen, back, buttocks
8.	Starting position: kneeling supported on hands<br>With an exhale, raise the bent right leg, with an inhale, lower it. After the series is finished, change sides.	12/ 2 series	Strengthening the buttocks
9.	Starting position: kneeling supported on hands<br>With an exhale, raise the bent leg to the side, with an inhale, return. After the series is complete, switch sides.	12/ 2 series	Strengthening the buttocks
10.	Starting position: kneeling supported on hands<br>With an inhale, straighten the right leg, with an exhale, bend and bring the knee to the chest,<br>we do not lower the right knee to the floor. After the series is finished, change sides.	12/ 2 series	Strengthening of abdominal muscles
11.	Starting position: kneeling supported on hands<br>With the exhale, tension, elevation of the pelvic floor muscles and raising the knees about 5 cm above the floor, with the inhale, relaxation of the pelvic floor muscles and lowering of the knees.	12/ 2 series	Strengthening the abdomen, back, buttocks

12.	Starting position: one-legged kneeling, right leg straightened to the side<br>With an inhale, lower the buttocks to the heel of the left leg, with an exhale, move to kneeling on one leg. After the series is finished, change sides.	12/ 2 series	Strengthening of the buttocks and thighs
13.	Starting position: lying on back, lower limbs bent<br>Right lower limb placed on left knee, right hand presses right knee towards left foot. Change sides.	Hold for 30 seconds	Gluteal stretch
14.	Starting position: lying on back, lower limbs bent<br>Raise straight right lower limb up. Change sides.	Hold for 30 seconds	Hamstring stretch
15.	Starting position: lying on back, lower limbs bent, feet connected with soles<br>With inhalation we direct air to the lower ribs and pelvic floor muscles, so as to feel their gentle lowering, with exhalation we feel the return movement of the ribs and gentle lifting of the pelvic floor muscles.	12	Pelvic floor relaxation
**Outline of the third meeting**			
1.	Starting position: standing position<br>Marching in place.	1 min	Preparation of the cardiovascular system
2.	Starting position: standing position<br>Pelvic movements – forward tilt (inhale) and backward tilt (exhale), then pelvic circulation.	12	Pelvic floor muscle activation and pelvic and spine mobilization
3.	Starting position: standing position, lower limbs hip-width apart<br>On inhale, move to squat, on exhale, return to the starting position.	12/ 2 series	Strengthening the gluteal muscles
4.	Starting position: standing position, lower limbs hip-width apart<br>With an exhale, move to a squat. With each subsequent exhale, move to a squat to the side, as much as there is enough space, then change sides.	12	Strengthening the gluteal muscles
5.	Starting position: standing position, lower limbs hip-width apart<br>With an exhale, abduct the right lower limb, with an inhale, return, after the completed series, change sides.	12/ 2 series	Strengthening the abdominal and gluteal muscles
6.	Starting position: standing position, lower limbs hip-width apart, torso slightly tilted forward<br>Exhale, raise the right lower limb back, inhale, return, after the series is complete change sides.	12/ 2 series	Strengthening the glutes, abdomen and back

7.	Starting position: standing position, lower limbs hip-width apart<br>With exhalation, tension and elevation of the muscles of the lower extremities, with inhalation, relaxation.	12/ 2 series	Activation of pelvic floor muscles
8.	Starting position: standing position, lower limbs hip-width apart<br>With an exhale, raise the right bent knee to the chest, with an inhale, lower it. After the series is finished, change sides.	12/ 2 series	Strengthening the abdominal muscles
9.	Starting position: standing position, lower limbs hip-width apart<br>With an exhale, move to a single-leg lunge, with an inhale, return, after the series is complete, change sides.	12/ 2 series	Strengthening the abdominal and gluteal muscles
10.	Starting position: standing position, lower limbs hip-width apart<br>With exhalation, activation of the pelvic floor muscles, raising the right bent knee to the chest and moving it to the side, with inhalation, lowering and relaxing the pelvic floor muscles. After the series is finished, change sides.	12/ 2 series	Strengthening the abdominal, pelvic floor and gluteal muscles
11.	Starting position: lying on back, lower limbs bent, right lower limb raised to a 90 degree angle at the hip and knee joints<br>With an exhale, raise the buttocks so that the pelvis does not twist, with an inhale, lower the buttocks. After the series is finished, change sides.	12/ 2 series	Strengthening the glutes, abdomen and back
12.	Starting position: kneeling supported on hands, right hand abducted<br>With exhalation, raise the straightened left lower limb, with inhalation, lower it. After the series is finished, change sides.	12/ 2 series	Strengthening the glutes, abdomen and back
13.	Starting position: lying on the right side, lower limbs bent, support on the right forearm<br>With an exhale, raise the torso, with an inhale, return, after the completed series, change sides.	12/ 2 series	Strengthening the glutes, abdomen and back
14.	Starting position: lying on back, lower limbs joined by feet lying on the floor<br>With inhalation we direct the breath to the pelvic floor muscles, feeling the relaxation, with exhalation we gently tense the pelvic floor muscles.	12/ 2 series	Pelvic floor relaxation
15.	Starting position: kneeling on heels<br>With exhalation, move to bending. Hold for a few breath cycles in this position.	1 min	Lumbar relaxation

Description of exercises conducted in the stationary group

**Outline of the first meeting**			
**No.**	**Position and move**	**Number of repetitions**	**Aim**
1.	Starting position: sitting on heels, hands placed on the lower, side ribs<br>With the inhale we direct the air to the hands, feeling their expansion, with the exhale we feel the return movement under the hands.	8	Diaphragm activation
2.	Starting position: sitting on heels, the right hand from the front embraces the lower ribs, the left hand is placed on the right shoulder<br>We direct the inhalation to the right hand to feel the expansion of the lower ribs, the hand placed on the shoulder is motionless.	5 by side	Diaphragm activation
3.	Starting position: kneeling<br>Arm circles backwards.	12	Mobilization and activation of the shoulder
4.	Starting position: kneeling<br>The upper limbs are bent at 90 degrees at the elbows, with inhalation the movement of abduction of the forearms, with exhalation the return of the forearms.	12	Strengthening the back muscles
5.	Starting position: kneeling<br>There is a resistance band in the hands, the right hand on the left shoulder, with an inhale we straighten the left upper limb at the elbow joint, with an exhale we flex it.	12	Strengthening the shoulder muscles
6.	Starting position: lying on back, lower limbs bent<br>With an exhale, perform the movement of bringing the pubic symphysis closer to the sternum (posterior tilt of the pelvis), with an inhale, return.	20	Activation of abdominal muscles
7.	Starting position: lying on back, lower limbs bent<br>A soft ball is placed between your knees, on the exhale the knees are squeezed, on the inhale relaxation.	12/ 2 series	Strengthening of the lower limbs, indirect activation of the pelvic floor muscles

8.	Starting position: lying on back, lower limbs bent<br>With an exhale, raise your buttocks, with an inhale, lower them.	12/ 2 series	Strengthening of the lower limbs, indirect activation of the pelvic floor muscles
9.	Starting position: lying on back, lower limbs bent<br>With the exhale, tension, elevation of the pelvic floor muscles, with the inhale, relaxation.	12/ 2 series	Direct activation of the pelvic floor muscles
10.	Starting position: lying on back, lower limbs bent<br>Resistance band wrapped above the knees, exhale to raise the buttocks and abduct the knees, inhale to return.	12/ 2 series	Strengthening the gluteal muscles
11.	Starting position: lying on the right side, lower limbs bent, support on the right forearm<br>With an exhale, raise the left knee, so as not to lift the foot, the torso is stabilized, with an inhale, lower the knee, after the series is completed, change sides.	12/ 2 series	Strengthening of the lower limbs, indirect activation of the pelvic floor muscles
12.	Starting position: lying on the right side, lower limbs bent<br>With an inhale, turn "behind the back", try to put the straight left arm behind you as far as possible, with an exhale, return, after the series is finished, change sides.	8	Stretching of the chest
13.	Starting position: lying on the right side, lower limbs extend<br>With an exhale, raise the left leg, with an inhale, lower it, after completing the series, change sides.	12/ 2 series	Strengthening of the lower limbs
14.	Starting position: lying on the right side, lower limbs bent, support on the right forearm<br>There is a soft ball under the left hand in front of the chest, with an exhale raise the torso and press the ball to the floor, with an inhale return, after the completed series change sides.	12/ 2 series	Torso strengthening
15.	Starting position: cross-legged position<br>Head tilt to the right, left, back and forth.	Hold for 30 seconds	Neck relaxation
16.	Starting position: cross-legged position<br>Bend the torso to the right, then to the left.	Hold for 30 seconds	Torso relaxation

17.	Starting position: cross-legged position<br>Turn the torso to the right, then to the left.	Hold for 30 seconds	Torso relaxation
**Outline of the second meeting**			
1.	Starting position: sitting on heels, hands placed on your lower, side ribs<br>With the inhale we direct the air to the hands, feeling their expansion, with the exhale under the hands we feel the return movement.	8	Diaphragm activation
2.	Starting position: sitting on heels, in hands a band that wraps around torso at the height of lower ribs<br>With an inhale we direct our breath to your lower ribs, the band resists, and with an exhale we feel the return movement and gently pull the ends of the band to hug your lower ribs tighter.	12	Diaphragm activation
3.	Starting position: kneeling supported on hands<br>With exhalation, the movement of approaching the pubic symphysis to the sternum, with inhalation, return.	12	Activation of the pelvic floor muscles and mobilization of the pelvis and spine
4.	Starting position: kneeling supported on hands<br>Pelvic rotation to the right, then change direction. When the pubic symphysis approaches the sternum, exhale, when it recedes, inhale.	12	Activation of the pelvic floor muscles and mobilization of the pelvis and spine
5.	Starting position: kneeling supported on hands<br>With exhalation, tighten and elevate the pelvic floor muscles, with inhalation, relax (strength approx. 30%).	12/ 2 series	Direct activation of the pelvic floor muscles
6.	Starting position: kneeling supported on hands, soft ball under right hand<br>Exhale, press hand into the ball, inhale, relax, after finished series, change sides.	12/ 2 series	Strengthening the abdominal muscles and shoulder girdle
7.	Starting position: neeling supported on hands, soft ball under feet<br>With exhalation, press the backs of the feet into the ball, with inhalation, relax.	12/ 2 series	Strengthening the abdominal muscles
8.	Starting position: lying on back, lower limbs bent, upper limbs raised up<br>Place a soft ball under right knee, between thigh and calf. With an exhale, bring right knee to chest and left hand to navel, with an inhale, return to the starting position. After the series is complete, change sides.	12/ 2 series	Strengthening the abdominal muscles
9.	Starting position: lying on back, lower limbs raised and bent in the knee and hip joints to 90 degrees, a ball placed between the knees	12/ 2 series	Strengthening the abdominal muscles

	With an exhale, abduct the right shin, with an inhale, return to the starting position, after the series is finished, change sides.		
10.	Starting position: kneeling supported on hands, right upper limb raised to the side<br>With exhalation, raise the straightened left lower limb, with inhalation, return. After finishing the series, change sides.	12/ 2 series	Strengthening of abdominal, back and gluteal muscles
11.	Starting position: kneeling supported on hands<br>With an exhale, raise the bent right leg, with an inhale, lower it. After the series is finished, change sides.	12/ 2 series	Strengthening the gluteal muscles
12.	Starting position: kneeling supported on hands<br>With an exhale, raise the bent leg to the side, with an inhale, return. After the series is finished, change sides.	12/ 2 series	Strengthening the gluteal muscles
13.	Starting position: kneeling supported on hands<br>With exhalation, tension, elevation of the pelvic floor muscles and raising the knees about 5 cm above the floor, with inhalation, relaxation of the pelvic floor muscles and lowering of the knees	12/ 2 series	Strengthening the abdominal muscles, pelvic floor, and back
14.	Starting position: lying on back, lower limbs bent<br>Right lower limb placed on left knee, right hand presses right knee towards left foot. Change sides.	Hold for 30 seconds	Glute stretching
15.	Starting position: lying on back, lower limbs bent, feet connected with soles<br>With inhalation we direct air to the lower ribs and pelvic floor muscles, so as to feel their gentle lowering, with exhalation we feel the return movement of the ribs and gentle lifting of the pelvic floor muscles.	12	Pelvic floor relaxation
**Outline of the third meeting**			
1.	Starting position: standing position<br>Marching in place.	1 min	Preparation of the cardiovascular system
2.	Starting position: standing position<br>Pelvic movements – forward tilt (inhale) and backward tilt (exhale), then pelvic circulation.	12	Pelvic floor muscle activation and pelvic and spine mobilization

3.	Starting position: standing position, lower limbs hip-width apart<br>On inhale, move to squat, on exhale, return to the starting position.	12/ 2 series	Strengthening the gluteal muscles
4.	Starting position: standing position, lower limbs hip-width apart, under the left foot is the end of the resistance band, the other end is held in the right hand<br>With an inhale, raise the right upper limb, with an exhale, lower it. After the series is finished, change sides.	12/ 2 series	Strengthening the abdominal muscles, pelvic floor, and back
5.	Starting position: sitting on a large ball<br>With the exhale, tension and elevation of the pelvic floor muscles, so as to feel the perineum area gently detaching itself from the surface of the ball, with the inhale, relaxation.	12/ 2 series	Activation of the pelvic floor muscles
6.	Starting position: sitting on a large ball<br>With an exhale, raise the right bent knee, with an inhale, lower it. After the series is finished, change sides.	12/ 2 series	Strengthening the abdominal muscles
7.	Starting position: lying on back, bent lower limbs are on the ball<br>With the exhale, raise the buttocks, with the inhale, lower them.	12/ 2 series	Strengthening of abdominal, back and gluteal muscles
8.	Starting position: lying on back, bent lower limbs are on the ball<br>With inhalation, rotation of the lower limbs and the ball to the right, with exhalation, return to the center. Change sides.	12/ 2 series	Strengthening the abdominal muscles
9.	Starting position: support with back, back resting on a large ball, lower limbs bent<br>With an exhale, raise the buttocks, with an inhale, lower them.	12/ 2 series	Strengthening of abdominal, back and gluteal muscles
10.	Starting position: kneeling supported on hands, right hand abducted lies on a large ball<br>With an exhale, raise the straightened left lower limb, with an inhale, lower it. After completing the series, change sides.	12/ 2 series	Strengthening of abdominal, back and gluteal muscles
11.	Starting position: standing, large ball held in both hands<br>With an inhale, move your right leg to a lunge to the side and transfer the ball to the right side. With an exhale, return to the starting position. After the series is complete, switch sides.	12/ 2 series	Strengthening of abdominal, back and gluteal muscles

12.	Starting position: standing position on the sensory disk<br>Anterior tilt (inhalation) and posterior tilt (exhalation) movements of the pelvis.	12/ 2 series	Improving balance and activating abdominal, buttock, back and pelvic floor muscles
13.	Starting position: standing position on the sensory disk<br>Transferring the body weight from heels to toes.	12/ 2 series	Improving balance and activating abdominal, buttock, back and pelvic floor muscles
14.	Starting position: kneeling on one leg, large ball in front, upper limbs touching the ball with hands<br>With exhalation, pelvic movement backward tilt, with inhalation forward tilt.<br>After the series is finished, change sides.	12	Relaxation of the lumbar spine
15.	Starting position: kneeling on heels, a large ball in front, hands on the ball<br>With an exhale, move to a bend. Hold this position for a few breath cycles.	1 min	Relaxation of the lumbar spine
